# Supplementary material for: Analysis of Microbiota and Mycobiota in Fungal Ball Rhinosinusitis: Specific Interaction between Aspergillus fumigatus and Haemophilus influenza?
Source: J Fungi (Basel). 2021 Jul 10;7(7):550. doi: 10.3390/jof7070550 (PMC8305266; doi:10.3390/jof7070550)

Table S1. Operative taxonomic units (OTUs) assigned to *Haemophilus, Pseudomonas, Staphylococcus, Streptococcus* and *Aspergillus* identified to the species level. Data presented in total read count and relative abundance among the genus.

	Reads count	(%)
Haemophilus	482,366	100
H. influenzae	416,443	86.3
Haemophilus sp.	65,923	13.6
Pseudomonas	185,542	100
P. aeruginosa	106,977	57.7
P. mosselii	43,574	23.4
P. migulae	29,039	15.6
P. synxantha	3,206	1.7
Pseudomonas sp.	2,746	1.7
Staphylococcus	202,784	100
S. aureus	186,534	91.9
S. lugdunensis	6,994	3.4
S. carnosus	4,860	2.4
S. epidermidis	4,229	2.1
S. caprae	175	0.08
S. saprophyticus	27	<0,01
Staphylococcus sp.	15	<0,01
Streptococcus	69,020	100
S. intermedius	36,994	53.6
S. pneumoniae	17,766	25.7
S. constellatus	14,144	20.5
S. sanguinis	60	0.1
S. dysgalactiae	46	0.1
Streptococcus sp.	10	<0.01
Aspergillus	1,075,193	100
Aspergillus section *Fumigati*	868,848	80.8
Aspergillus section *Flavi*	165,693	15.4
Aspergillus section *Nidulantes*	40,365	3.7
Aspergillus sp.	287	0.03

Figure S1. Fungal richness and diversity calculated based on ITS1 and ITS2. Alpha, inverse Simpson, Shannon and Simpson are displayed.

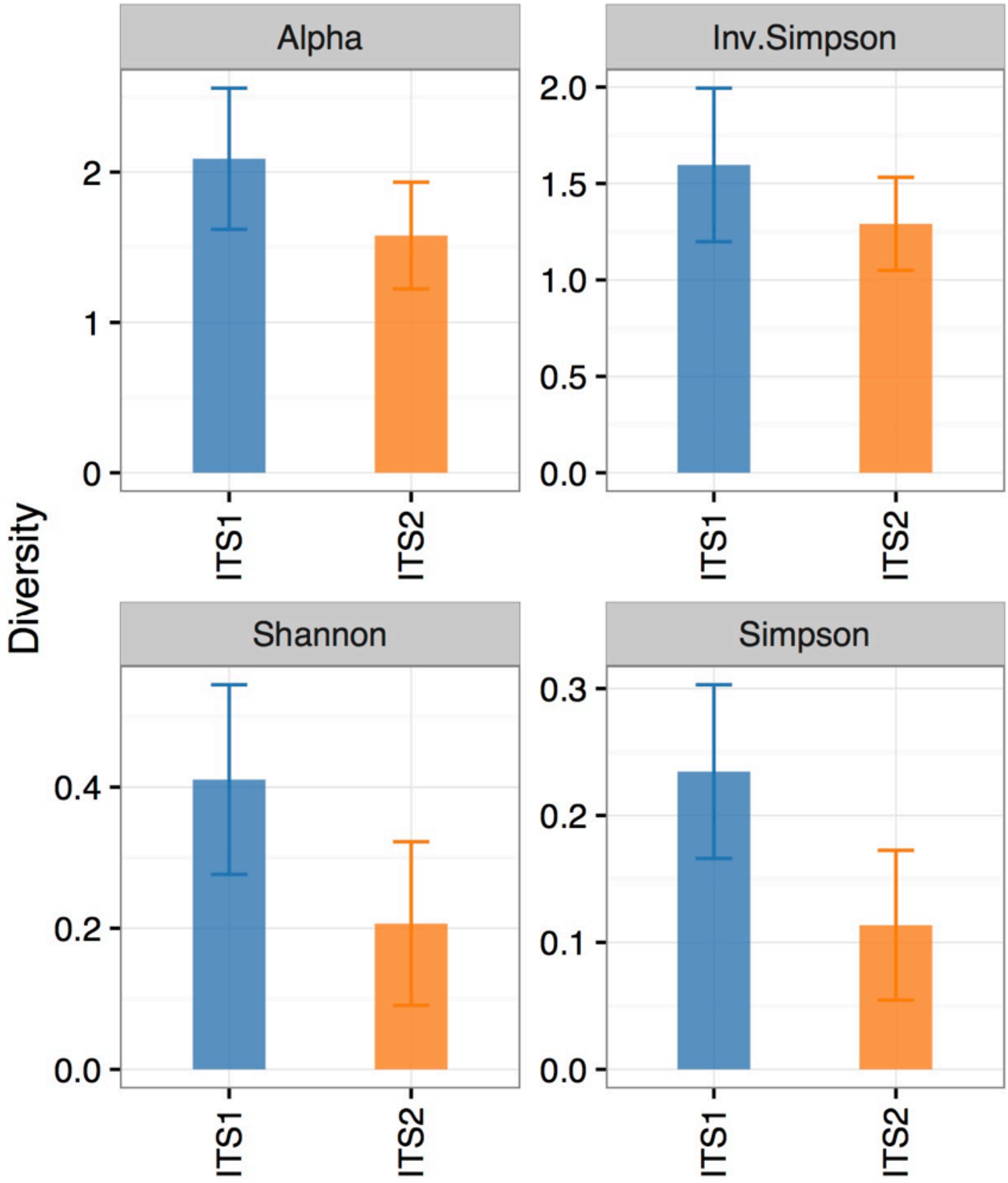

Figure S2: Heatmap of the relative abundance of major* (A) bacterial and (B) fungal taxa among the 45 samples analysed through 16S and ITS2 targeted metagenomic analysis respectively.

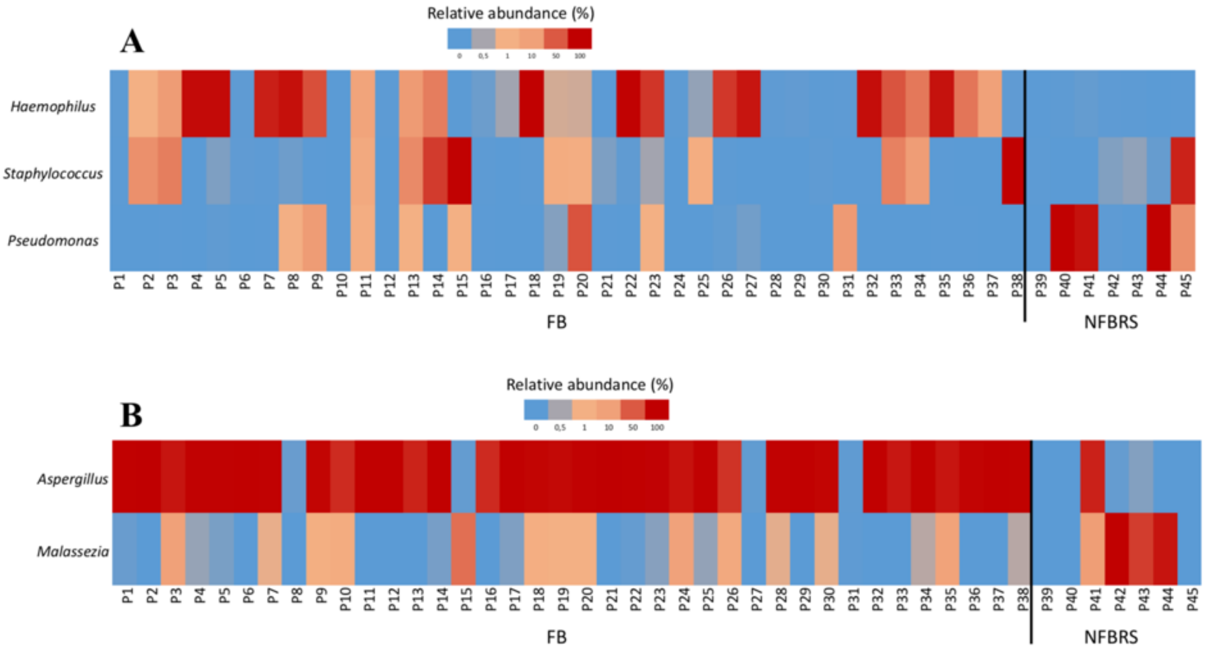

*major bacterial and fungal taxa were considered if taxa was >1% relative abundance in >25% samples. FB: fungal balls; NFBRS: Non-fungal ball rhinosinusitis

Figure S3. Bacterial diversity represented in number of reads and relative abundance for all taxa found

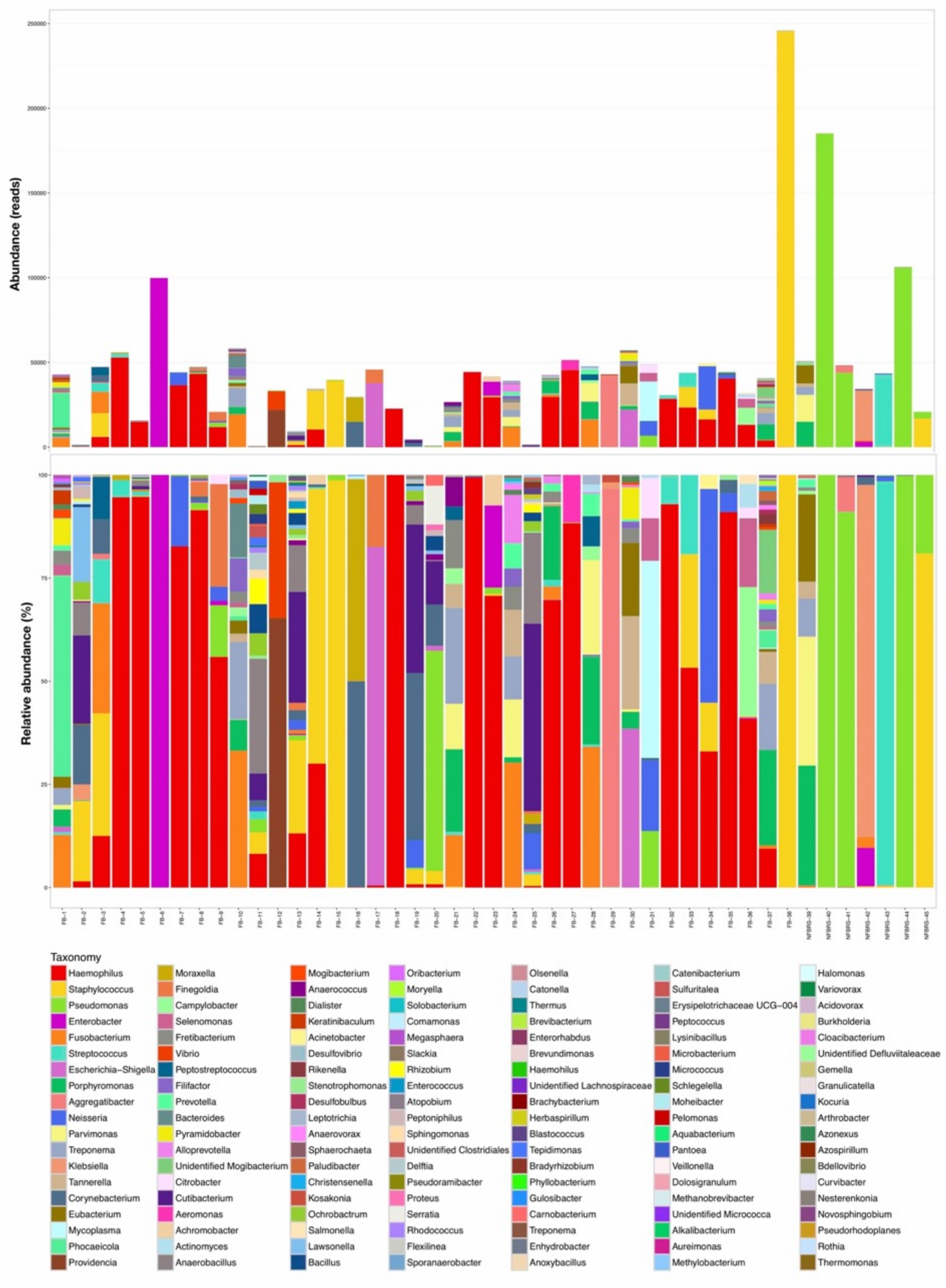

Figure S4. Fungal diversity represented in number of reads and relative abundance for all taxa found

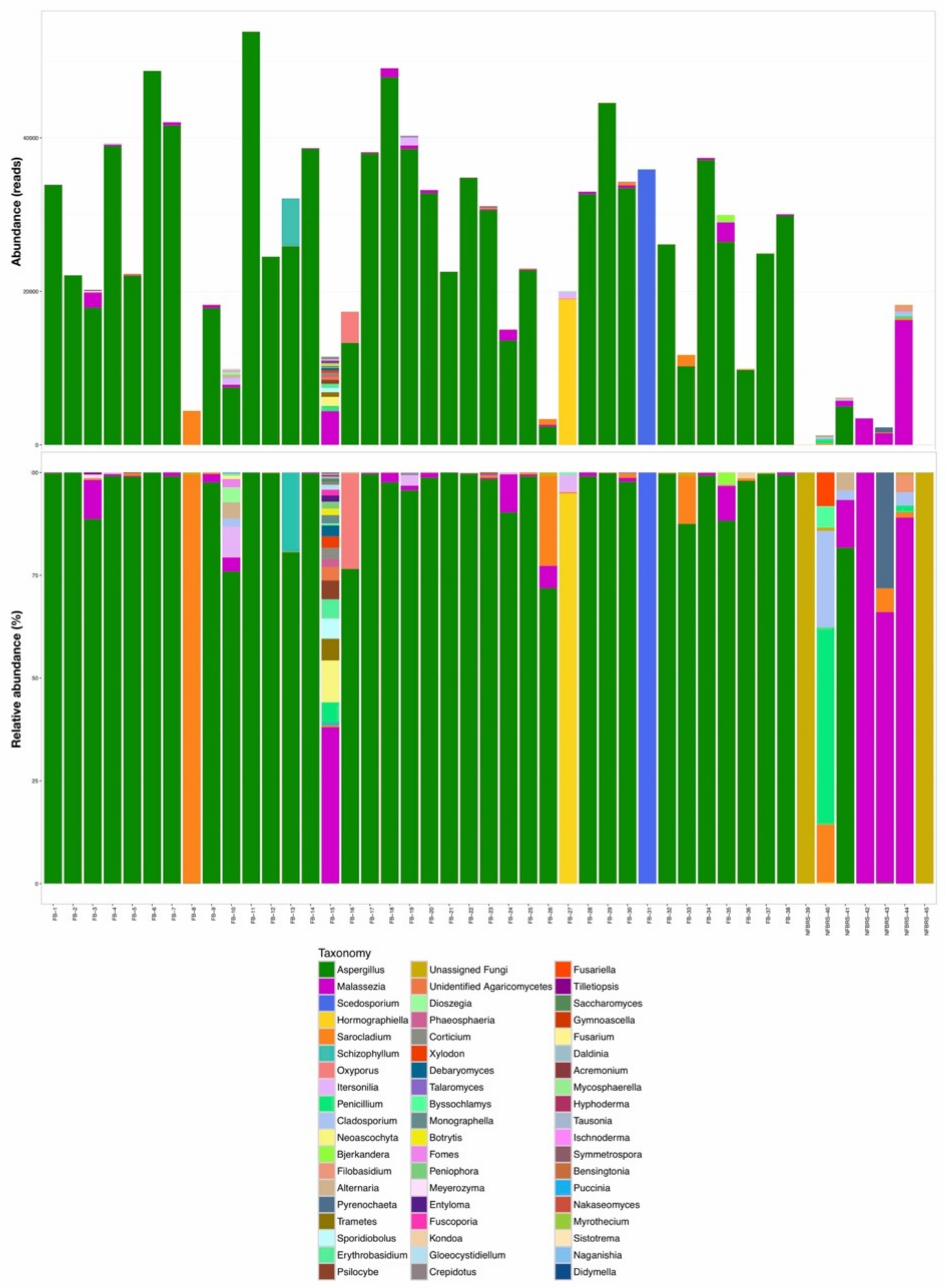

Supplement: Supplementary file 1 [file jof-07-00550-s001.zip › jof-1224643-supplementary.pdf]
